# IL-10-producing CD4^+^ T cells negatively regulate fucosylation of epithelial cells in the gut

**DOI:** 10.1038/srep15918

**Published:** 2015-11-02

**Authors:** Yoshiyuki Goto, Aayam Lamichhane, Mariko Kamioka, Shintaro Sato, Kenya Honda, Jun Kunisawa, Hiroshi Kiyono

**Affiliations:** 1 Division of Mucosal Immunology, Department of Microbiology and Immunology, Institute of Medical Science, The University of Tokyo, Tokyo, Japan; 2 Department of Medical Genome Sciences, Graduate School of Frontier Sciences, The University of Tokyo, Chiba, Japan; 3 Core Research for Evolutional Science and Technology (CREST), Japan Science and Technology Agency, Tokyo, Japan; 4 Laboratory of Vaccine Materials, National Institutes of Biomedical Innovation, Health and Nutrition (NIBIOHN), Osaka, Japan; 5 Graduate School of Medicine and Faculty of Medicine, University of Tokyo, Tokyo, Japan; 6 RIKEN Center for Integrative Medical Sciences (IMS), Kanagawa, Japan; 7 International Research and Development Center for Mucosal Vaccines, Institute of Medical Science, The University of Tokyo, Tokyo, Japan; 8 Graduate School of Pharmaceutical Sciences and Graduate School of Dentistry, Osaka University, Osaka, Japan; 9 Department of Microbiology and Infectious Diseases, Kobe University Graduate School of Medicine, Kobe, Japan; 10 Deapartment of Immunology, Graduate School of Medicine, Chiba University, Chiba, Japan; 11 Division of Molecular Immunology, Medical Mycology Research Center, Chiba University

## Abstract

Fucosylated glycans on the surface of epithelial cells (ECs) regulate intestinal homeostasis by serving as attachment receptors and a nutrient source for some species of bacteria. We show here that epithelial fucosylation in the ileum is negatively regulated by IL-10-producing CD4^+^ T cells. The number of fucosylated ECs was increased in the ileum of mice lacking T cells, especially those expressing αβ T cell receptor (TCR), CD4, and IL-10. No such effect was observed in mice lacking B cells. Adoptive transfer of αβTCR^+^ CD4^+^ T cells from normal mice, but not IL-10-deficient mice, normalized fucosylation of ECs. These findings suggest that IL-10-producing CD4^+^ T cells contribute to the maintenance of the function of ECs by regulating their fucosylation.

The mammalian gastrointestinal tract is colonized by a community of bacteria^1^, and the host establishes physical, chemical, and immunological barriers as a shield to limit the exposure to these bacteria^2^,^3^. As the first barrier in the intestine, many different subsets of epithelial cells (ECs) reside in the intestinal epithelial monolayer. These subsets include absorptive enterocytes, goblet cells, Paneth cells, enteroendocrine cells, and antigen-sampling M cells^2^. Several lines of evidence point to the fact that both host-derived factors (e.g., cytokines and chemokines) and gut environmental factors (e.g., commensal bacteria, dietary products, and their metabolites) affect the intestinal barrier function^2^. For example, luminal bacteria induce the secretion of anti-microbial proteins (e.g., regenerating islet–derived protein 3γ) by ECs; this secretion limits bacterial load on the intestinal epithelium^4^,^5^.

The surfaces of ECs bear a coating (the glycocalyx) consisting of various glycoproteins and glycolipids, and ECs also secrete a large amount of glycosylated mucins, which act as a protective barrier in the intestine^6^. In addition to its protective function, the glycocalyx on the ECs also provides attachment sites for commensal bacteria, as exemplified by the attachment of *Lactobacillus* to glycolipids or glycoproteins^7^,^8^, and pathogens, as seen in *Helicobacter pylori* attachment to fucosylated or sialylated glycans^9^. Moreover, certain species of commensal and pathogenic bacteria have evolved to utilize the glycosylated molecules produced by the ECs^10^. Fucose is a residual sugar frequently present at the termini of glycoconjugates in the intestinal epithelium^3^,^11^. Some indigenous bacteria preferentially induce fucosylation of the intestinal epithelium^12^, and some reports, including ours, have proposed that fucosylation of intestinal ECs provides a niche for a stable microbial ecosystem^13^,^14^.

Mammals possess multiple sets of fucosyltransferases that mediate fucosylation through the transfer of guanosine-diphosphate fucose to acceptor molecules including oligosaccharides, glycoproteins, and glycolipids^15^,^16^. In the intestine, fucosylation is achieved by the addition of α(1,2)-fucose to terminal galactose residues by fucosyltransferase-1 (Fut1) and fucosyltransferase-2 (Fut2)^15^,^16^. It was reported that ECs in the small intestine selectively express the *Fut1* and *Fut2* genes: M cells of the Peyer’s patches express *Fut1*, whereas goblet cells and enterocytes express *Fut2*^17^,^18^. The expression of *Fut1* seems to be constitutive, whereas the expression of *Fut2* can be induced by environmental stimuli and stresses, such as bacterial colonization^18^,^19^. Experiments with germ-free mice have demonstrated that Fut2-mediated α(1,2)-fucosylation was induced after weaning together with the appearance of commensal bacteria^12^. In addition, colonization by a single type of commensal bacteria, such as segmented filamentous bacteria *Bacteroides thetaiotaomicron* and *Bacteroides fragilis*, was sufficient to induce Fut2-mediated epithelial fucosylation^12^,^14^,^20^,^21^. Recently, mutations in *Fut2* have been shown to be associated with inflammatory and autoimmune diseases such as Crohn’s disease and type 1 diabetes^22^,^23^, suggesting the involvement of host immune cells in the regulation of fucosylation. We have recently reported that IL-22 produced by type 3 innate lymphoid cells is critical for the induction and regulation of epithelial fucosylation. In the present study, we show that IL-10-producing CD4^+^ T cells play a pivotal role in the negative regulation of epithelial fucosylation in the intestine.

## Results

### T cell–deficient mice have increased numbers of fucosylated epithelial cells (F-ECs) with increased *Fut2* expression in the intestine

**Figure 1:**
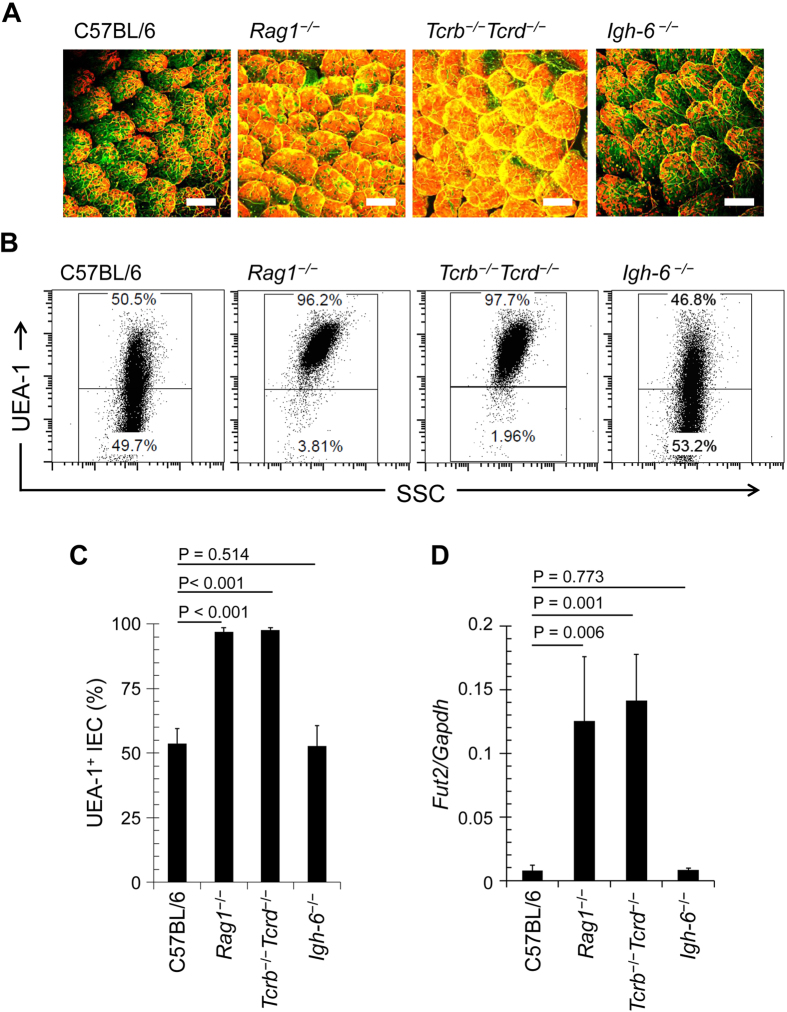
T cell deficiency enhances epithelial fucosylation in the ileum. (**A**) Whole-mount staining of the ileum of C57BL/6, *Rag1*^−/−^, *Tcrb*^−/−^*Tcrd*^−/−^, and *Igh-6*^−/−^ mice was performed with UEA-1 (red) for α-1,2 fucose staining and wheat germ agglutinin (green) for epithelial cell counterstaining. Scale bars: 100 μm. Data are representative of 4 independent experiments. (B,C) Ileal ECs were stained with CD45 antibody and UEA-1. CD45^−^ cells were gated and analyzed for UEA-1 binding by flow cytometry. Representative dot plots are shown in (**B**). Mean percentages ± s.d. (*n* = 6) of UEA-1^+^ ECs are shown in (**C**). (**D**) Quantitative PCR analysis of *Fut2* expression in ileal ECs. Data normalized against the expression of *Gapdh* are shown as mean ± s.d. (*n* = 6 from 2 independent experiments).

In this study, we focused on the ileum for the analysis of epithelial fucosylation because duodenal ECs have minimal fucosylation, whereas ileal ECs are highly fucosylated^14^. To assess whether epithelial fucosylation is affected by lymphocytes, we examined epithelial fucosylation in the ileum of recombinase-activating gene-1–deficient mice (*Rag1*^−/−^) and *scid* mice that lack mature T and B cells^24^,^25^. Higher numbers of fucosylated ECs (F-ECs) were found on the ileal epithelium of both *Rag1*^−/−^ and *scid* mice than on that of control mice (Fig. 1A–C and Supplementary Figure 1A). In line with these findings, the increase of F-ECs in *Rag1*^−/−^ and *scid* mice was accompanied by increased *Fut2* mRNA expression in ileal ECs (Fig. 1D and Supplementary Figure 1B).

We then aimed to determine whether T or B cells are responsible for the regulation of epithelial fucosylation. To address this question, we examined βδ T cell receptor (TCR) knockout (*Tcrb*^−/−^*Tcrd*^−/−^) mice, which lack T cells, and *Igh-6*^−/−^ mice, which lack B cells. The number of F-ECs and *Fut2* mRNA expression were not significantly altered in ileal ECs of *Igh-6*^−/−^ mice but were increased in *Tcrb*^−/−^*Tcrd*^−/−^ mice (Fig. 1A–D), suggesting that T cells are pivotal in the epithelial fucosylation in the ileum.

### CD4^+^ effector T cells expressing αβTCR are critical for the regulation of epithelial fucosylation in the intestine

**Figure 2:**
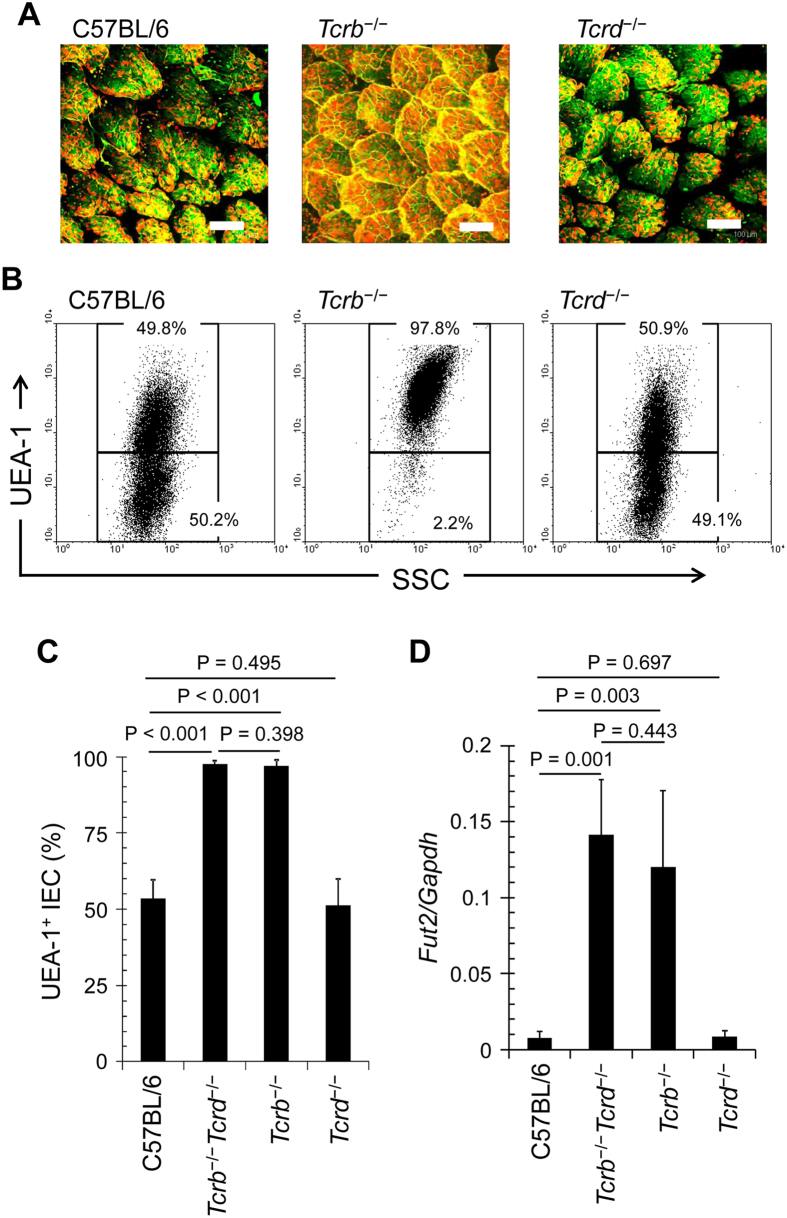
αβT cells regulate fucosylation of ileal ECs. (**A**) Whole-mount staining of the ileum of C57BL/6, *Tcrb*^−/−^, and *Tcrd*^−/−^ mice was performed with UEA-1 (red) and wheat germ agglutinin (green). Scale bars: 100 μm. Data are representative of 4 independent experiments. (**B,C**) Ileal ECs were stained with CD45 antibody and UEA-1. CD45^−^ cells were gated and analyzed for UEA-1 binding by flow cytometry. Representative dot plots are shown in (**B**). Mean percentages of UEA-1^+^ ECs are shown ± s.d. (n = 6) in (**C**). (**D**) Quantitative PCR analysis of *Fut2* expression in ileal ECs. Data normalized against the expression of *Gapdh* are shown as mean ± s.d. (*n* = 6 from 2 independent experiments).

The intestine contains T cells expressing either αβTCR (αβT cells) or γδTCR (γδT cells)^26^. To determine whether epithelial fucosylation is regulated by αβT cells, γδT cells, or both, we analyzed epithelial fucosylation in the ileum of *Tcrb*^−/−^ and *Tcrd*^−/−^ mice. Like *Rag1*^−/−^ and *scid* mice, *Tcrb*^−/−^ mice showed increased numbers of F-ECs and *Fut2* mRNA expression, whereas these parameters were similar in *Tcrd*^−/−^ and control mice (Fig. 2A–D), suggesting that the regulation of epithelial fucosylation in the ileum is solely mediated by αβT cells. Athymic *nu/nu* mice also had a high number of F-ECs and increased *Fut2* expression in ileal ECs in comparison with control mice (Supplementary Figure 2A,B). These data imply that thymus-derived αβT cells downregulate *Fut2* expression and associated epithelial fucosylation in the ileum.

**Figure 3:**
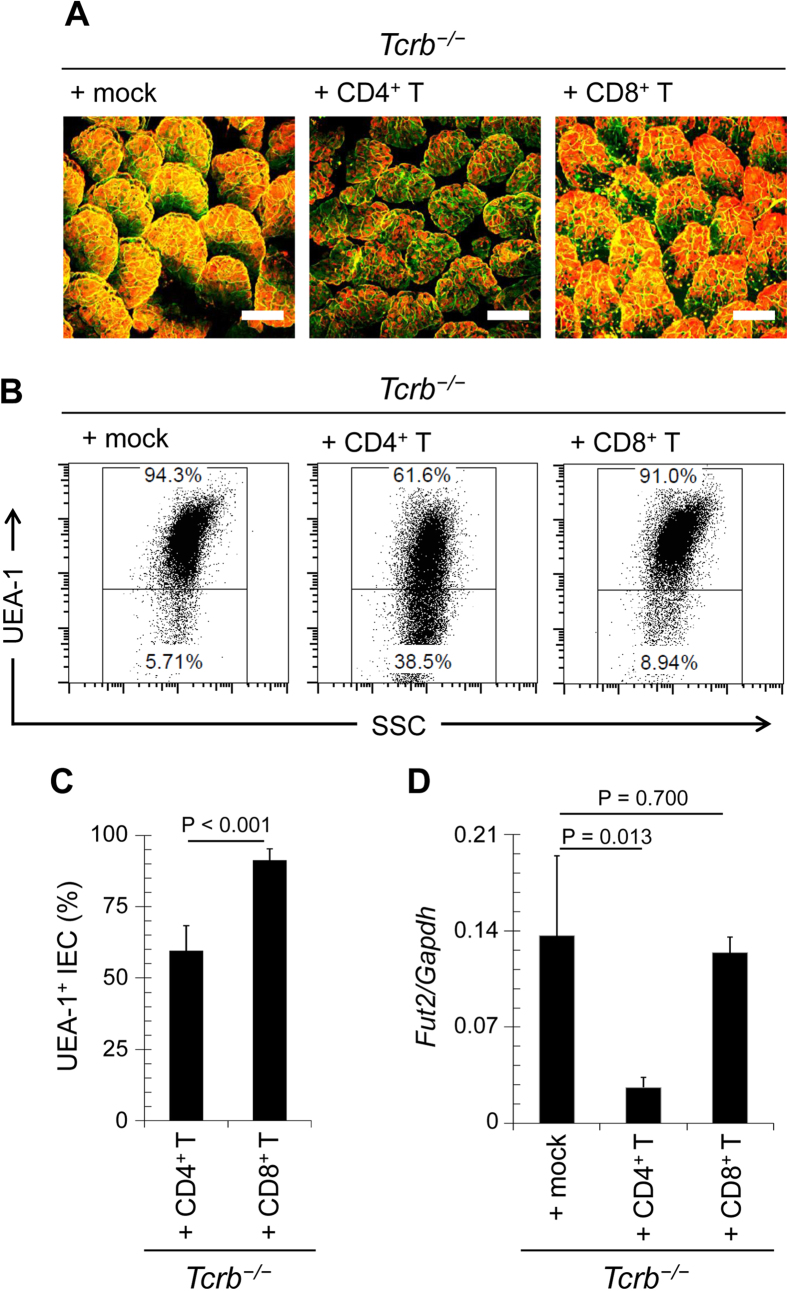
Epithelial fucosylation of ECs is downregulated by CD4^+^ T cells. CD4^+^ or CD8^+^ T cells were purified from the spleens of C57BL/6 mice and adoptively transferred into *Tcrb*^−/−^ mice. After 4 weeks, ileal ECs were analyzed for fucosylation by whole-mount staining (**A**) and flow cytometry (**B**,**C**), and for *Fut2* expression by quantitative PCR (**D**). Scale bars in (**A**): 100 μm. Data in (**A**,**B**) are representative of 6 independent experiments. Data in (**C**,**D**) are shown as mean ± s.d. (*n* = 6 from 2 independent experiments).

The αβT cells express either CD4 or CD8. To identify the sub-population of T cells responsible for the regulation of epithelial fucosylation, we adoptively transferred purified CD4^+^ or CD8^+^ T cells into *Tcrb*^−/−^ mice. The number of F-ECs was reduced in mice that received CD4^+^ T cells, but not CD8^+^ T cells, in comparison with the number in mock-treated *Tcrb*^−/−^ mice (Fig. 3A–C). *Fut2* mRNA expression in ECs was also decreased by adoptive transfer of CD4^+^ T cells (Fig. 3D). These results indicate that epithelial fucosylation is negatively regulated by CD4^+^ αβT cells.

### IL-10-producing CD4^+^ T cells negatively regulate F-ECs

**Figure 4:**
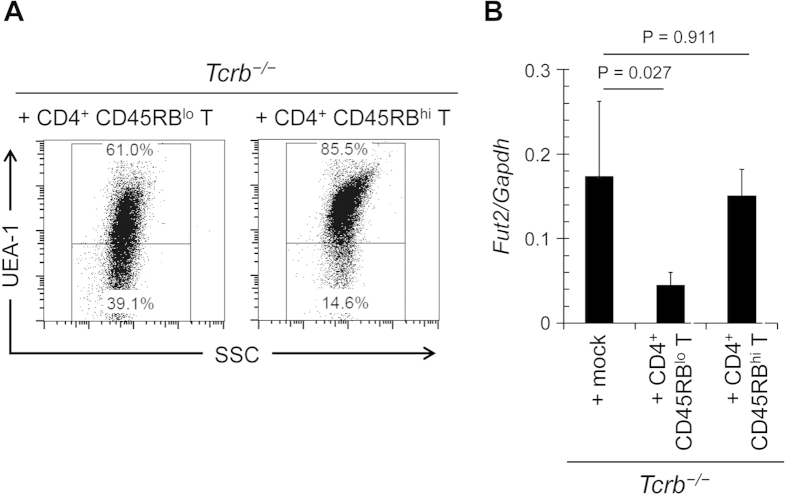
Effector T cells are involved in the downregulation of epithelial fucosylation in the ileum. CD4^+^ CD45RB^hi^ or CD4^+^ CD45RB^lo^ T cells were purified from the spleens of C57BL/6 mice and adoptively transferred into *Tcrb*^−/−^ mice. After 4 weeks, ileal ECs were analyzed for fucosylation by flow cytometry (**A**) and for *Fut2* expression by quantitative PCR (**B**). Data in (**A**) are representative of 6 independent experiments. Data in (**B**) are shown as mean ± s.d. (*n* = 6 from 2 independent experiments).

We then examined whether naïve CD4^+^ T cells or effector CD4^+^ T cells downregulate epithelial fucosylation. To address this question, we isolated CD4^+^CD45RB^lo^ effector T cells and CD4^+^CD45RB^hi^ naïve T cells from normal mice. Adoptive transfer of CD4^+^CD45RB^lo^ effector T cells, but not CD4^+^CD45RB^hi^ naïve T cells, into *Tcrb*^−/−^ mice down-regulated epithelial fucosylation and decreased the expression of *Fut2* mRNA in ileal ECs (Fig. 4A,B), suggesting that effector CD4^+^ T cells are responsible for the reduction of epithelial fucosylation.

**Figure 5:**
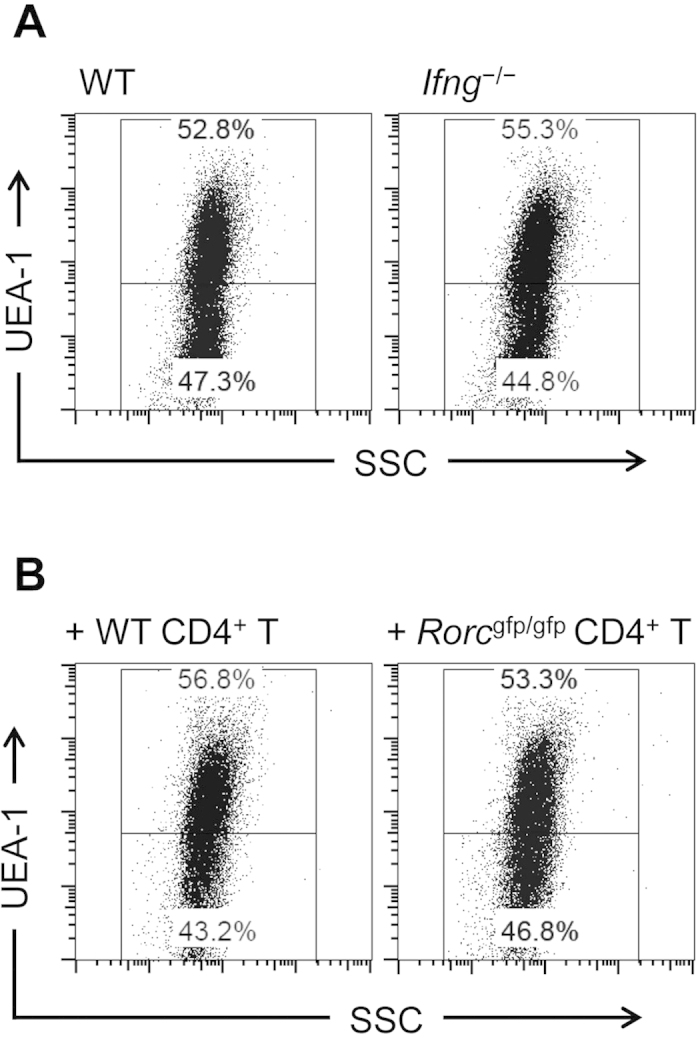
IFN-γ and IL-17 are not involved in the regulation of ileal EC fucosylation. (**A**) Ileal ECs from wild-type (WT) and *Ifng*^−/−^ C57BL/6 mice were analyzed for UEA-1 binding. The data are representative of 3 independent experiments. (**B**) CD4+ T cells were purified from spleens of WT and *Rorc*^*gfp/gfp*^ C57BL/6 mice and adoptively transferred into *Tcrb*^−/−^ mice. Ileal ECs were isolated 4 weeks after transfer and used for the analysis of UEA-1 binding. The data are representative of 4 individual experiments.

The intestinal effector CD4^+^ T cells produce various cytokines such as interferon γ (IFN-γ), IL-17, and IL-10^27^. In *Ifng*^−/−^ mice, epithelial fucosylation was not affected (Fig. 5A). Additionally, adoptive transfer of CD4^+^ T cells from *Rorc*^*gfp/gfp*^ mice (which fail to develop the IL-17-producing Th17 population^28^) or from wild-type (WT) mice had the same effect on epithelial fucosylation in *Tcrb*^−/−^ mice; the F-EC number was still reduced upon adoptive transfer of the Th17-deficient CD4^+^ T cell population (Fig. 5B). Thus, it is likely that neither IFN-γ nor IL-17 is involved in the inhibition of epithelial fucosylation in the ileum.

**Figure 6:**
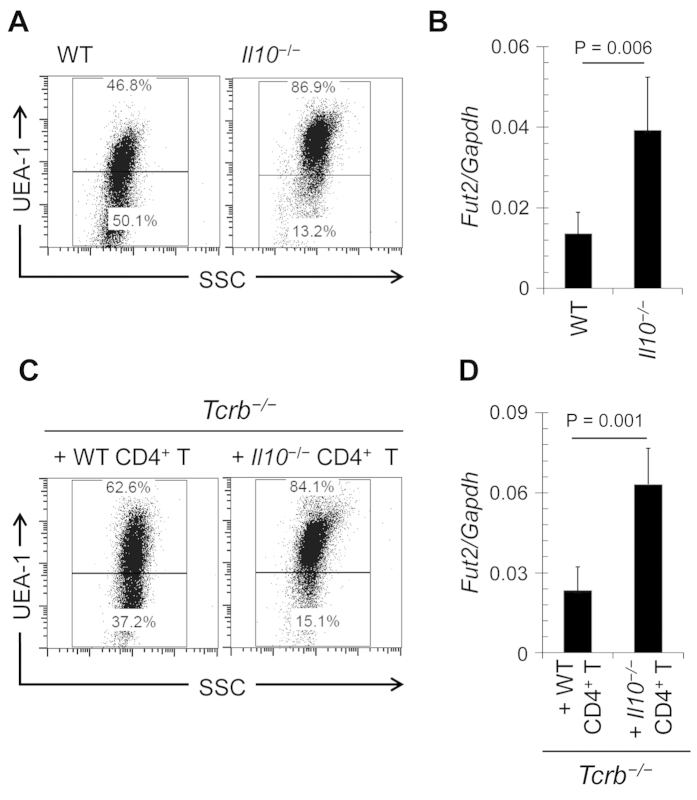
IL-10 derived from CD4^+^ T cells regulates epithelial fucosylation. (**A**) Flow cytometric analysis of the ileum of wild-type (WT) and *Il10*^−/−^ mice was performed with UEA-1. (**B**) Quantitative PCR analysis of *Fut2* expression in ileal ECs of WT and *Il10*^−/−^ mice. (**C,D**) CD4^+^ T cells were purified from the spleens of WT and *Il10*^−/−^ mice and adoptively transferred into *Tcrb*^−/−^ mice. After 4 weeks, ileal ECs were analyzed for fucosylation by flow cytometry (**C**) and for *Fut2* expression by quantitative PCR (**D**). Data in (A**,C**) are representative of 6 independent experiments. Data in (**B**,**D**) are shown as mean ± s.d. (*n* = 6 from 2 independent experiments).

These results led us to focus on the possible involvement of IL-10-producing T cells^29^ in downregulation of epithelial fucosylation. The number of F-ECs and *Fut2* mRNA expression were increased in the ileum of IL-10-deficient (*Il10*^−/−^) mice in comparison with WT mice (Fig. 6A,B). These parameters were also increased upon treatment of WT mice with anti-IL-10 receptor–neutralizing antibody (Supplementary Figure 3).

To determine whether CD4^+^ T cells were the major source of IL-10, we analyzed *Il10* expression in different subsets of immune cells in the ileum of WT mice. We found that *Il10* mRNA expression in CD4^+^ T cells was higher than that in other major immune cell populations (CD8^+^ T cells, IgA^+^ plasma cells, eosinophils, macrophages, and dendritic cells) (Supplementary Figure 4A). IL-10 production in ileal CD4^+^ T cells was confirmed in IL-10^Venus^ reporter mice (Supplementary Figure 4B). These findings allowed us to determine whether IL-10 produced by CD4^+^ T cells was responsible for the downregulation of epithelial fucosylation. CD4^+^ T cells were purified from *Il10*^−/−^ and WT mice and adoptively transferred into *Tcrb*^−/−^ mice. The ability to downregulate epithelial fucosylation in *Tcrb*^−/−^ mice was weaker in the case of *Il10*^−/−^ CD4^+^ T cells than in the case of WT CD4^+^ T cells (Fig. 6C,D). These results suggest that IL-10-producing CD4^+^ T cells play an important role in the downregulation of epithelial fucosylation in the intestine.

## Discussion

Several lines of evidence including ours suggested that commensal bacteria and type 3 innate lymphoid cells are a prerequisite for the induction of epithelial fucosylation^12^,^13^,^14^. Our current study extends our knowledge of the control of epithelial fucosylation by demonstrating its negative regulation by IL-10-producing CD4^+^ T cells.

In the intestine, several regulatory cell types maintain homeostasis by preventing excessive inflammatory responses; these cells recognize intestinal antigens including commensal bacteria^30^. IL-10 orchestrates intestinal immune homeostasis by controlling T cells^31^, macrophages^32^, and ECs^33^, including goblet cells^34^. Our current finding suggests an additional role for IL-10-producing CD4^+^ T cells in the regulation of *Fut2* expression and consequently epithelial fucosylation. Among T cells, Foxp3^+^ Treg cells and CD4^+^ Foxp3^−^ Tr1 cells are predominant in the gut and produce IL-10^35^,^36^,^37^. Under some conditions, IL-10 is also produced by other CD4^+^ T cells (e.g., Th1 cells)^38^. Although adoptive transfer of IL-10-producing CD4^+^ T cells decreased the frequency of F-ECs in T cell–deficient mice, intraperitoneal administration of recombinant IL-10 alone did not affect the frequency of F-ECs in the ileum (Supplementary Figure 5). Therefore, it is plausible that IL-10 is necessary but not sufficient for the downregulation of epithelial fucosylation and that some additional molecules on the surface of CD4^+^ T cells and/or produced by these cells are required to maintain appropriate levels of epithelial fucosylation. Our current efforts are aimed at revealing which types of CD4^+^ T cells and what additional molecules are pivotal for the IL-10-mediated maintenance of epithelial fucosylation.

Fucose is involved in maintaining microbiota homeostasis in the mucosal epithelia^14^,^39^,^40^. Fucose moieties expressed on ECs and secreted into the lumen are used by commensal bacteria as nutrients. For example, *Bacteroides* species produce enzymes to cleave fucose from host glycans and use it as a nutrient^41^,^42^. Genetic changes in the ability to synthesize fucose are associated with altered gut microbial composition^40^,^43^. In addition to commensal bacteria, inactivating mutations in the *Fut2* gene reduce susceptibility to human Norwalk virus, which uses α1,2-fucosylated glycans as receptors^44^. Changes in susceptibility to other pathogens (e.g., *Helicobacter pylori*^45^, *Candida albicans*^46^, and *Streptococcus pneumoniae*^47^) have been reported.

The role of glycans in ECs as a physical barrier between the host and microbes is also highlighted by findings in mice lacking functional core 1–derived O-glycans^48^. These mice show rapid induction of severe spontaneous colitis with progressive severity, possibly initiated by defective barrier function of the mucosa and greater translocation of bacteria into the mucosal tissue^48^. Similarly, mice lacking core 2–derived O-glycans show a baseline defect in intestinal permeability and are more susceptible to intestinal challenge with dextran sodium sulfate^49^. Although the role of fucose on ECs in intestinal inflammation is not clearly understood, recent genome-wide association studies have implicated *Fut2* in the pathogenesis of many intestinal inflammatory diseases including Crohn’s disease in the human population^22^. Of note, αβTCR mutant mice and *Il10*^−/−^ mice develop spontaneous intestinal inflammation^50^,^51^,^52^. The fact that epithelial fucosylation is highly upregulated in *Tcrb*^−/−^ and *Il10*^−/−^ mice implies that fucosylation is at least partly related to intestinal inflammation. In fact, *Il10*^−/−^ mice with colonic inflammation are reported to show increased expression of the H antigen, which is a product of the *Fut2* activity, on the colonic epithelium^53^. Enhanced expression of *Fut2* and subsequent increase in the proportion of F-ECs in the ileum may lead to the development of intestinal inflammation.

The mechanism by which IL-10 regulates *FUT2* expression still needs to be determined. Our experiments show that both IL-10 receptor (IL-10R) subunits, α and β, are expressed only in a fraction of F-ECs (Supplementary Figure 6). Although the functional difference between IL-10R^+^ and IL-10R^−^ F-ECs remains unclear, this finding suggests that IL-10 acts directly on IL-10R^+^ ECs. An interesting possibility is that additional molecules expressed on the surface and/or produced by CD4^+^ T cells convert IL-10R^−^ ECs or ECs positive for only one IL-10R chain into ECs expressing both types of IL-10R. In ECs, *FUT2* expression is induced by stimulation of ERK–ATF2 and JNK–c-Jun signaling^13^. IL-10 inhibits the p38/MAPK-activated protein kinase-2 pathway^54^, which activates ATF2^55^. A similar mechanism may inhibit glycosylation and may be responsible for IL-10-mediated inhibition of *Fut2* expression and associated fucosylation of ECs.

## Methods

### Mice

C57BL/6, Balb/c, C.B-17/lcr-*scid/scid*, and Balb/c-*nu/nu* mice were purchased from CLEA (Japan). *Ifng*^−/−^, *Igh-6*^−/−^, *Il10*^−/−^, *Rag1*^−/−^, *Rorc*^*gfp/gfp*^, *Tcrb*^−/−^, and *Tcrd*^−/−^ mice (all in the C57BL/6 background) were purchased from The Jackson Laboratory. The *Il10*^*Venus*^ mice were generated as previously reported^56^. All animals were maintained in the experimental animal facility at the University of Tokyo, and the experiments were approved by the Animal Care and Use Committee of the University of Tokyo and conducted in accordance with the committee’s guidelines.

### Whole-mount immunofluorescence staining

Whole-mount immunofluorescence staining was performed as previously described^14^,^18^. Briefly, the mucus layer was removed by flushing the ileum with phosphate-buffered saline, and then tissues were fixed in 4% paraformaldehyde. The fixed tissues were stained with fluorescence-labeled *Ulex europaeus* agglutinin I (UEA-1; Vector Laboratories) and wheat germ agglutinin (Invitrogen). Images were analyzed by using a confocal laser-scanning microscope (Leica TCS SP2).

### Cell preparations

ECs were prepared as previously described^14^,^18^. Briefly, after removal of Peyer’s patches, the intestine was opened longitudinally and washed extensively several times with ice-cold phosphate-buffered saline. The intestine was then cut into 1-cm pieces, which were incubated in 1 mM ethylenediaminetetraacetic acid in phosphate-buffered saline at 37 °C for 15 min with gentle shaking. The suspension was passed through a 70-μm cell strainer, and ECs obtained were washed with Dulbecco’s modified Eagle’s medium containing 20% fetal calf serum.

To isolate mononuclear cells, tissues remaining after EC isolation were finely minced, stirred 3 times in RPMI-1640 containing 2% fetal calf serum and 1 mg/ml collagenase (Wako) for 20 min and centrifuged on a discontinuous Percoll (GE Healthcare) gradient (40% and 75%)^57^. Cells at the interface between the 40% and 75% fractions were collected.

To prepare single-cell suspensions of splenocytes, excised spleen fragments were pressed through a 70-μm cell strainer using the plunger end of a syringe. Collected cells were treated with lysis buffer (0.15 M NH_4_Cl, 10 mM KHCO_3_, 0.1 mM ethylenediaminetetraacetic acid) to remove red blood cells and washed with 2% fetal calf serum in RPMI-1640.

### Flow cytometry and cell sorting

Isolated ileal ECs were stained with anti-mouse CD45 antibody (clone 30-F11; BioLegend) to distinguish between hematopoietic and non-hematopoietic cells. To exclude non-viable cells, Via-Probe cell-viability solution (BD Biosciences) was used. Staining with anti-mouse EpCAM antibody (clone G8.8; eBioscience) was used to confirm that viable CD45^−^ cells were ileal ECs. UEA-1 was used to assess fucosylation; non-stained samples served as controls.

Mononuclear cells were pre-incubated with anti-CD16/32 (Fcγ RII/III) antibody (BD Biosciences) and stained with fluorescence- or biotin-labeled antibodies. The following antibodies were used: anti-mouse CD45, anti-mouse CD4 (clone RM4–4; BD Biosciences), anti-mouse CD8α (clone 53–6.7; BD Biosciences), anti-mouse CD3ε (clone 145–2C11; BD Biosciences), anti-mouse CD45R (B220; clone: RA3–6B2, BD Biosciences), anti-mouse CD45RB (clone 16A; BD Biosciences), anti-mouse CD11b (clone M1/70; BD Biosciences), anti-mouse CD11c (clone HL3; BD Biosciences), anti-mouse IgA (clone mA-6E1; eBioscience), anti-mouse F4/80 (clone BM8; eBioscience), anti-mouse CD25 (clone PC61; BioLegend), anti-mouse TCRβ (clone H57–597; BD Biosciences), anti-mouse γδ TCR (clone GL3; BD Biosciences), anti-mouse FR4 (clone 12A5; BioLegend), anti-mouse IL-10Rα (clone 3F9; BD Biosciences), and anti-mouse IL-10Rβ (clone 547324; R&D Systems). Cells stained with biotinylated antibodies were further stained with fluorescence-labeled streptavidin (BD Biosciences). Staining with isotype antibodies was carried out. To isolate specific subsets of T cells from splenocytes, B220^+^ B cells were initially depleted by magnetic-activated cell sorting using B220 microbeads (Miltenyi). Flow cytometric analysis and cell sorting were performed by using FACS Canto and FACS Aria systems (BD Biosciences), respectively. The purity of isolated cells was consistently >95%.

### Adoptive transfer experiments

The sorted cells (1 × 10^6^) were administered intraperitoneally to 4–6-week-old *Tcrb*^−/−^ mice. Recipient mice were analyzed by whole-mount staining and flow cytometry 4 weeks after adoptive transfer. Reconstitution of adoptively transferred cells to the spleen and small intestine was confirmed by flow cytometry at the time of analysis.

### Anti-IL-10 receptor antibody and recombinant IL-10 treatment

Mice were intraperitoneally injected with 250 μg of purified anti-IL-10 receptor monoclonal antibody (clone 1B1.3A; eBioscience) or purified rat IgG1 as isotype control on every third day 4 times. Two days after the final injection, mice were used for the analysis of epithelial fucosylation.

Mice were intraperitoneally injected with 4 μg of recombinant IL-10 (BioLegend) in 100 μL of PBS or with vehicle alone^57^,^58^. Injections were performed on days 0, 2, 4, 6, and 8, and epithelial fucosylation was analyzed on day 10.

### Quantitative PCR analysis

Cells were lysed in TRIzol (Invitrogen), and total RNA was extracted according to the manufacturer’s instructions. Purified RNA was reverse-transcribed by using a SuperScript VILO cDNA Synthesis Kit (Invitrogen). Quantitative PCR was carried out on a Lightcycler II system (Roche Diagnostics) with primers and probe combinations designed by using the Roche Universal Probe Library Assay Design Center (Roche), and the mRNA levels were presented as the ratios of target mRNAs to the internal control, glyceraldehyde-3-phosphate dehydrogenase (*Gapdh*) mRNA. The following primers and probes were used: *Gapdh* (sense 5′-tgtccgtcgtggatctgac, antisense 5′-cctgcttcaccaccttcttg; probe #80), *Fut2* (sense 5′-gcggttcgtccattccta, antisense 5′-aaaggtacctgggcactcg, probe #76), and *Il10* (sense 5′-cagagccacatgctcctaga, antisense 5′-gtccagctggtcctttgttt; probe #41).

### Statistical analysis

Statistical analyses were conducted by using Microsoft Excel and GraphPad PRISM. Results were compared by using two-tailed Student’s *t*-tests. Data were plotted as means ± standard deviation (s.d.).

## Additional Information

**How to cite this article**: Goto, Y. *et al.* IL-10-producing CD4^+^ T cells negatively regulate fucosylation of epithelial cells in the gut. *Sci. Rep.* **5**, 15918; doi: 10.1038/srep15918 (2015).

## Supplementary Material

Supplementary Information
