# Supplementary material for: IL-10-producing CD4+ T cells negatively regulate fucosylation of epithelial cells in the gut
Source: Sci Rep. 2015 Nov 2;5:15918. doi: 10.1038/srep15918 (PMC4629126; doi:10.1038/srep15918)
Supplement: Supplementary Information [file srep15918-s1.pdf]

Supplementary Information

IL-10-producing $CD4^+$ T cells negatively regulate fucosylation of epithelial cells in the gut

Yoshiyuki Goto[1,3,+], Aayam Lamichhane[1–4,+], Mariko Kamioka[1,3–5], Shintaro Sato[1,3], Kenya Honda[3,6], Jun Kunisawa[1,4,7–9,*], Hiroshi Kiyono[1–3, 5,7,10,*]

[1] Division of Mucosal Immunology, Department of Microbiology and Immunology, Institute of Medical Science, The University of Tokyo, Tokyo, Japan

[2] Department of Medical Genome Sciences, Graduate School of Frontier Sciences, The University of Tokyo, Chiba, Japan

[3] Core Research for Evolutional Science and Technology (CREST), Japan Science and Technology Agency, Tokyo, Japan

[4] Laboratory of Vaccine Materials, National Institutes of Biomedical Innovation, Health and Nutrition (NIBIOHN), Osaka, Japan

[5] Graduate School of Medicine and Faculty of Medicine, University of Tokyo, Tokyo, Japan

[6] RIKEN Center for Integrative Medical Sciences (IMS-RCAI), Kanagawa, Japan

[7] International Research and Development Center for Mucosal Vaccines, Institute of Medical Science, The University of Tokyo, Tokyo, Japan

[8] Graduate School of Pharmaceutical Sciences and Graduate School of Dentistry, Osaka University, Osaka, Japan

[9] Department of Microbiology and Infectious Diseases, Kobe University Graduate School of Medicine, Kobe, Japan

[10] Deapartment of Immunology, Graduate School of Medicine, Chiba University, Chiba, Japan.

[+] These authors contributed equally.

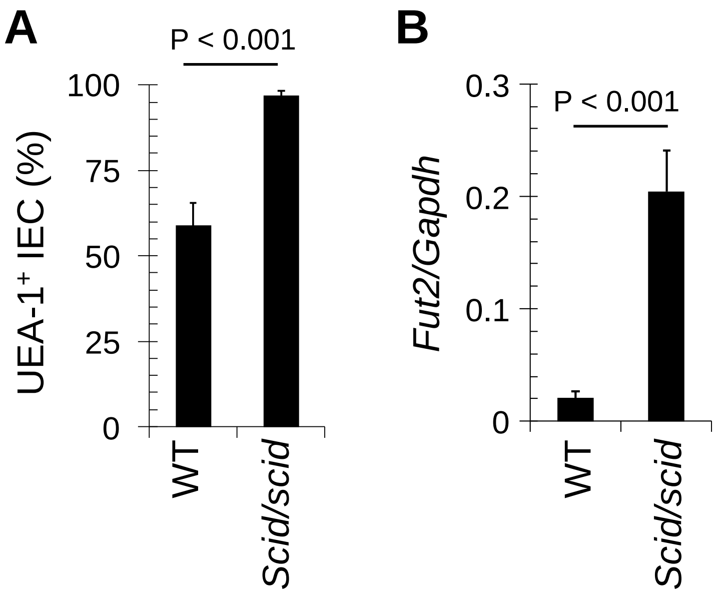

Supplementary Figure 1. *Scid* mice have highly upregulated fucosylation and *Fut2* expression in ileal ECs.
(**A**) Mean percentage of UEA-1$^+$ ECs in the ileum of wild-type (WT) and *scid* mice. (**B**) *Fut2* expression analyzed by quantitative PCR in ileal ECs of WT and *scid* mice. Data normalized against the expression of *Gapdh* are shown as mean $\pm$ s.d. (n = 5).

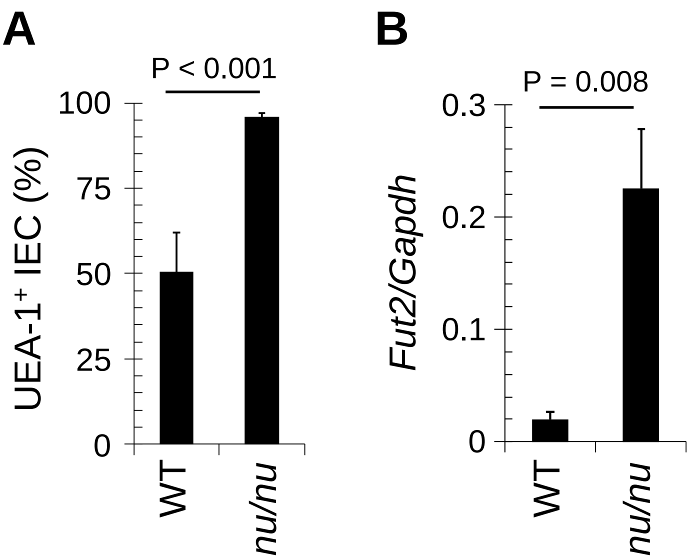

Supplementary Figure 2. Athymic *nude* mice have highly upregulated fucosylation and *Fut2* expression in ileal IECs.
(**A**) Mean percentage of UEA-1^+ ECs in the ileum of wild-type (WT) and athymic nude (*nu/nu*) Balb/c mice. (**B**) *Fut2* expression analyzed by quantitative PCR in ileal ECs of WT and *nu/nu* mice. Data normalized against the expression of *Gapdh* are shown as mean ± s.d. (n = 5).

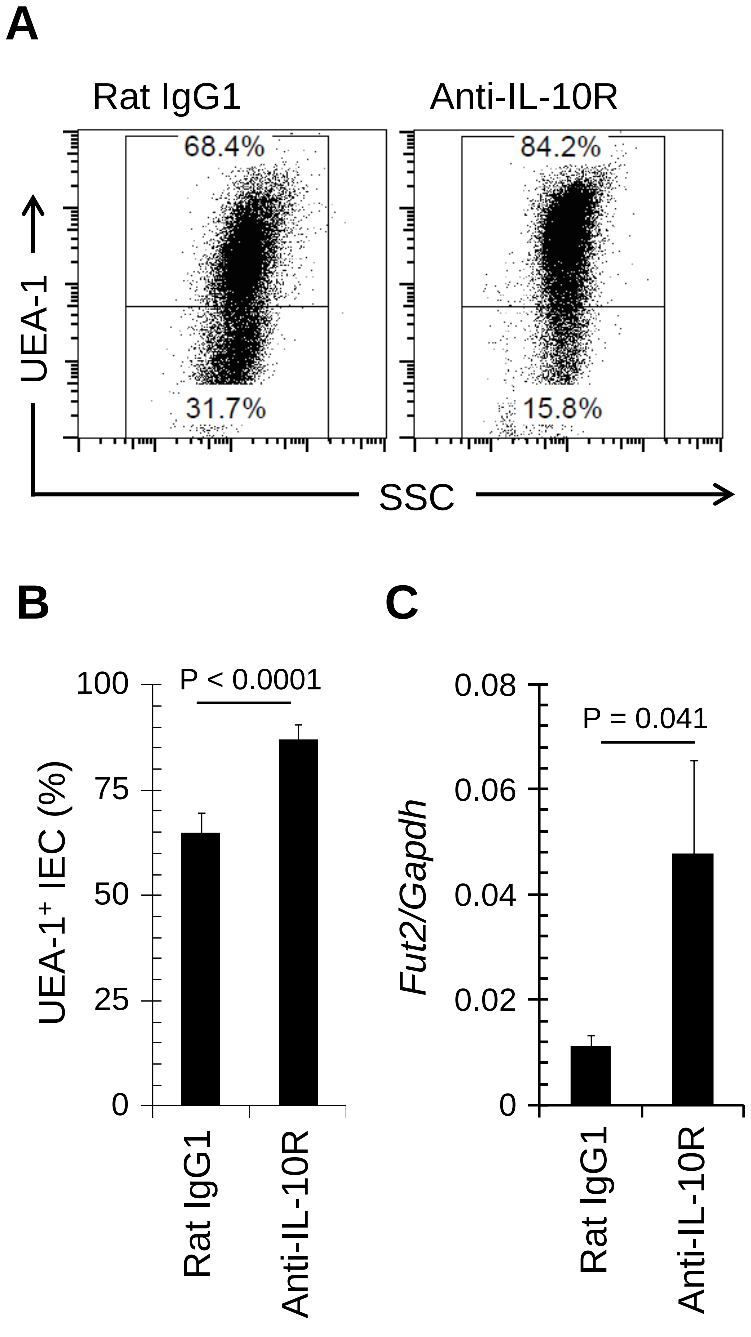

Supplementary Figure 3. IL-10 receptor blockage increases epithelial fucosylation. C57BL/6 mice received 250 μg of rat IgG1 or anti-mouse IL-10 receptor (anti-IL-10R) antibody on every third day 4 times. Two days after the final administration, IECs were analyzed for UEA-1 binding. Representative flow cytometry dot plots (**A**), mean percentages of UEA-1^{+} IECs (**B**), and quantitative PCR analysis of *Fut2* expression (**C**) are shown. Quantitative PCR data normalized against the expression of *Gapdh* are shown as mean ± s.d (n = 4 from 2 independent experiments).

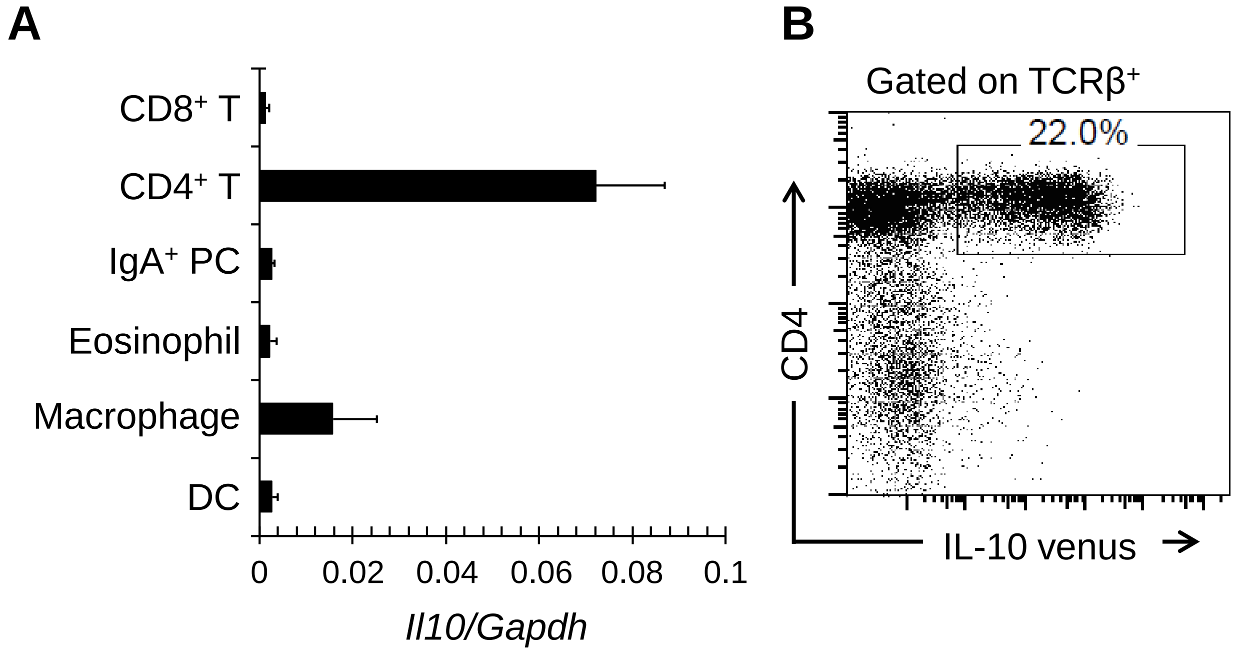

Supplementary Figure 4. CD4$^+$ Tr1 cells are major producers of IL-10 in the ileum.
(**A**) CD8$^+$ T cells, CD4$^+$ T cells, IgA$^+$ plasma cells (PC), eosinophils, macrophages, and dendritic cells (DC) were purified from the ileum of C57BL/6 mice and their *Il10* mRNA expression was analyzed by quantitative PCR. The data normalized against the expression of *Gapdh* are shown as mean ± s.d. (**B**) Flow cytometric analysis of IL-10-producing T cells in the ileum of IL-10^{venus} mice. Similar results were obtained in 3 individual experiments.

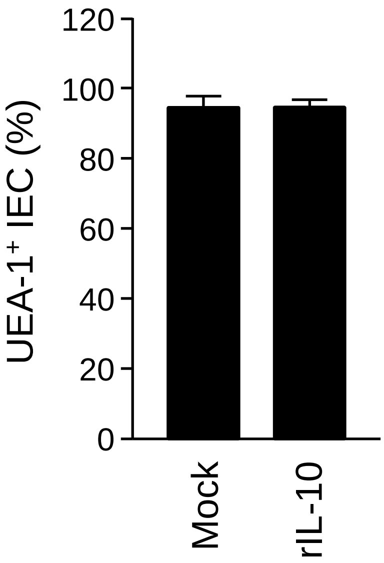

Supplementary Figure 5. IL-10 alone is not sufficient for the down-regulation of epithelial fucosylation.
T-cell-deficient *nu/nu* mice were intraperitoneally treated with recombinant IL-10 every other day 5 times. Two days after the last administration, epithelial fucosylation was examined by flow cytometry. Percentages of UEA-1^{+} IECs are shown. Data are shown as mean ± s.d (n = 8 from 2 independent experiments).

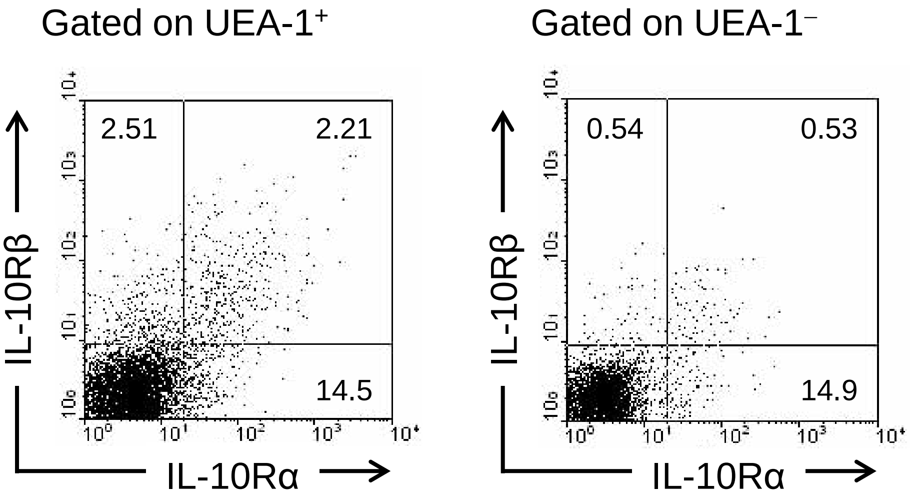

Supplementary Figure 6. IL-10 receptor expression on ECs. ECs were isolated from the ileum of C57/BL6 mice for the analysis of IL-10 receptor α and β expression on UEA-1^{+} (left) and UEA-1^{-} (right) epithelial cells. Similar results were obtained in 3 individual experiments.
